# The role of Effortful Control in Pychopathology Amongst Older Psychiatric Patients

**DOI:** 10.1192/j.eurpsy.2022.175

**Published:** 2022-09-01

**Authors:** X. Brancart, E. Dierckx, G. Rossi, R. De Raedt

**Affiliations:** 1 Vrije Universiteit Brussel, Department Of Psychology, Personality And Psychopathology Research Group (peps), Brussels, Belgium; 2 Vrije Universiteit Brussel (& Alexianen Zorggroep Tienen, psychiatric hospital), Department Of Psychology, Brussel, Belgium; 3 Ghent University, Department Of Experimental Clinical And Health Psychology, Ghent, Belgium

**Keywords:** Transdiagnostic, temperament-based personality types, reactive temperament, regulative temperament

## Abstract

Background and aim: Based upon a person-centred approach, there is a growing interest in neurobiological transdiagnostic factors, such as reactive temperament (Behavioral Inhibition (BIS) and Behavioral Activation Systems (BAS)) and regulatory processes (Effortful Control (EC)). Three temperament-based personality types (Resilient, Undercontrolled and Overcontrolled type (RUO)) have been replicated in adolescents and younger adults with several clinical problems; with the resilient type (highest scores on EC) consistently showing less psychological symptoms. However, these RUO types have not been studied yet in older adults with mental disorders. Therefore, the current study investigates (1) whether these RUO types can be replicated based on the aforementioned reactive and regulative temperamental factors in older inpatients and (2) whether a higher EC is related to the presence of less psychopathology. Methods: The EC, BIS/BAS, SCL-90-R and ADP-IV questionnaires were administered to 96 older patients (<= 60 years) admitted to a psychiatric hospital. Results: Cluster analysis resulted in a solution of three atypical types: a Resilient type (low BIS, mean BAS, high EC), a Dysregulated type (high BIS, high BAS, low EC) and an “Apathic” type (mean BIS, low BAS, mean EC). Comparison of means revealed that the Resilient type has the highest scores on EC and shows less clinical symptoms and maladaptive personality traits. Discussion: Of the 3 RUO types, only the Resilient type was fully replicated in older psychiatric patients. Strengthening EC might be useful as an additional therapy in order to reduce clinical symptoms, possibly leading to a better treatment outcome.

**Disclosure:**

No significant relationships.

